# Complete Genome Sequence and Annotation of the *Staphylococcus aureus* Strain HG001

**DOI:** 10.1128/genomeA.00783-17

**Published:** 2017-08-10

**Authors:** Isabelle Caldelari, Béatrice Chane-Woon-Ming, Céline Noirot, Karen Moreau, Pascale Romby, Christine Gaspin, Stefano Marzi

**Affiliations:** a Université de Strasbourg, CNRS, Architecture et Réactivité de l’ARN, UPR 9002, Strasbourg, France; b UBIA & PF GenoToul Bioinfo, INRA, Castanet Tolosan, France; c Université Claude Bernard Lyon 1, Ecole Normale Supérieure de Lyon, INSERM U1111, CNRS UMR 5308, CIRI, Lyon, France

## Abstract

*Staphylococcus aureus* is an opportunistic Gram-positive pathogen responsible for a wide range of infections from minor skin abscesses to life-threatening diseases. Here, we report the draft genome assembly and current annotation of the HG001 strain, a derivative of the RN1 (NCT8325) strain with restored *rbsU* (a positive activator of SigB).

## GENOME ANNOUNCEMENT

*Staphylococcus aureus* is an opportunistic Gram-positive pathogen that is present in one-third of the population on skin and in nostrils (1) as commensal bacteria, but it is also responsible for a wide range of infections from minor skin abscesses to life-threatening diseases, such as septicemia and toxic shock syndrome (2). *S. aureus* represents a major health problem due to the insurgence of antibiotic-multiresistant strains in hospital and community settings.

Many complete *S. aureus* genome sequences are available in the NCBI database (currently 116). These genomes were obtained from isolates from patients with sepsis and also from laboratory strains or have been generated with molecular tools. Several transcriptomic analyses have been conducted under various conditions by different laboratories (3–8) using the standard model HG001 strain, a derivative of the RN1 (NCT8325) strain with repaired *rbsU* (a positive activator of SigB). The strain still contains a mutation at *tcaR*, a transcriptional regulator involved in teicoplanin susceptibility and biofilm (9). Nevertheless, a complete genome sequence of this commonly used *S. aureus* laboratory strain is not available. Here, we present the draft genome sequence of the reference strain HG001.

Chromosomal DNA was extracted using the Gentra Puregene kit (Qiagen) following the manufacturer’s guidelines. Whole-genome sequencing was conducted with the PacBio RS platform (GATC, Biotech SARL, Germany). Two single-molecule real-time (SMRT) cells were used to sequence the genome (mean coverage of 78 times the genome), which was assembled *de novo* from 6 contigs with the SMRT portal (PacBio). The pseudomolecule was constructed by referring to the NCTC8325 genome sequence (GenBank accession no. CP000253, NCBI reference sequence no. NC_007795). The Seaview tool (10) has been used to manually align contigs. The final genome assembly consists of a 2,819,767-bp-long DNA molecule. Genome annotation has been done using Prokka v1.10 (11), which predicted 2,617 coding sequences (CDS), 62 tRNAs, 15 rRNAs, 1 transfer-messenger RNA (tmRNA), and 59 other noncoding RNAs. This annotation has been manually curated, with the addition of 4 more peptides and 418 noncoding RNAs from the Staphylococcal Regulatory RNAs Database (http://srd.genouest.org/raw//sRNA/sRNA_NCTC8325.gff) (12) and is available for download (http://www-ibmc.u-strasbg.fr/spip-arn/spip.php?rubrique152).

### Accession number(s).

The complete genome sequence for the HG001 strain has been deposited in GenBank under the accession no. CP018205.
